# The Use of Smart Devices for Mental Health Diagnosis and Care

**DOI:** 10.3390/jcm11185359

**Published:** 2022-09-13

**Authors:** Ziv Lautman, Shahar Lev-Ari

**Affiliations:** 1 Department of Bioengineering, Stanford University, Stanford, CA 94305, USA; 2 Department of Genetics, Stanford University, Stanford, CA 94305, USA; 3 Department of Health Promotion, School of Public Health, Sackler Faculty of Medicine, Tel-Aviv University, Tel-Aviv 69978, Israel

**Keywords:** mental health, smartphone, wearable, diagnosis, care

In 2019, more than 970 million people worldwide suffered from a mental disorder, with anxiety and depressive disorders as the leading culprits [1]. In 2020, due to the COVID-19 pandemic, initial estimates showed a 26% and 28% increase in anxiety and major depressive disorders, respectively [1]. Of those suffering from mental disorders, 70% did not receive adequate psychological care or achieve clinical remission, mainly due to the inaccessibility of care [1,2]. With more than 2.3 billion people using smartphones and other smart-wearables worldwide [2], smart devices have the potential to reduce the barrier of entry to medical care for mental disorders. These devices show viability in expanding diagnostic capabilities, preventative treatment, and long-term therapies in clinical practice. This editorial explores some promising and exciting research in enabling equitable and cost-effective care for mental disorders using smart devices.

Traditionally, the clinical diagnosis and research of various mental health disorders are based on the retrospective self-reports of patients. However, these tools are not able to capture the major depressive disorder (MDD) dynamics or generate insights into the temporal variability of anxiety and mood symptoms [2,3]. Moreover, self-reports are affected by recall bias [2]. The Ecological Momentary Assessment (EMA) is a digital, real-time version of the traditional clinical assessment that patients can repeatedly answer with their smart devices, in a natural setting. The EMA correlates with the data collected using traditional methods [4], and in depression, it has already led to new insights about emotion reactivity, cortisol patterns, and daily rumination [2]. As described by Colombo et al., the EMA enables the continuous monitoring of patient’s symptoms, thus allowing clinicians to (i) early identify symptoms’ worsening, (ii) preserve continuous communication with their patients, (iii) longitudinally track treatment efficacy, and (iv) foresee momentary mood changes [2].

The potential of EMA use in clinical practice is extended with the integration of the physiological data (e.g., heart rate, sleep, exercise, etc.) gathered from wearable devices and the behavioral data (e.g., social media activity, number of calls per day, etc.) gathered via smartphones, allowing for a multimodal approach [2,4]. Analyzing both the physiological and behavioral data unlocks an event-based sampling method that could enhance patient-specific models powered by machine and deep learning methods. Hence, collecting the EMA, physiological, and behavioral data using smart devices can support the building of predictive models for a patient’s future stress level or mood [2].

With predictive models in hand, smart devices can suggest care and interventions to patients. Like the EMA, the Ecological Momentary Intervention (EMI) is delivered directly on smart devices, in real-time, throughout the day, and without a face-to-face meeting with a clinician. For example, in depression, three EMI features were found to be effective: (i) obtaining visual feedback on daily diagnostics, (ii) access to educational material on depression, and (iii) option to have direct communication with a clinician [2].

Several studies using the EMA, physiological, or behavioral data with smart devices have already produced promising results in identifying digital biomarkers for MDD characterization and diagnosis [4]. In addition, the EMA and EMI have provided psychological support to anxiety- and stress-related disorders [2,3,4]. Furthermore, smart devices have shown exciting potential in various clinical settings besides mental health. This includes the measurement of motor parameters derived from wearable devices in multiple sclerosis [5]; multisite photoplethysmography technology for blood pressure assessment [6], and the use of EMI in reducing pain among patients suffering from chronic pain, especially in the long term [7]. 

Around the world, billions of people are using smart devices such as smartphones and wearable devices. With recent advances in predictive models and the use of EMA and EMI techniques, those devices hold the potential to serve as personal psychiatric diagnostic and care tools, providing access to care in real time, whenever needed [4]. The real-time diagnosis of stress, anxiety, or depression, followed by immediate care and intervention, could dramatically impact patients worldwide at a fraction of the traditional cost of treatment. It is important to note that smart devices are not intended to replace traditional care; on the contrary, both serve as complements to each other—digital tools providing clinicians with consistent, longitudinal data from patients and clinicians providing medically backed treatments and therapies via digital tools. Finally, with the recent advances and cost reduction in multiomics, integrating the longitudinal tracking of psychometrics (using EMA), physiology (using smart devices), and biology (using multiomics) could open a new frontier in revealing the biomarkers, including digital ones, for mental health disorders.
